# Study on Low-Temperature and Fatigue Performance of High RAP Content Hot Recycled Asphalt Mixture Based on the Degree of Blending (DOB)

**DOI:** 10.3390/polym14214520

**Published:** 2022-10-25

**Authors:** Jianming Wu, Hao Sun, Lixia Wan, Jiangang Yang, Shuyi Wang

**Affiliations:** 1 Jiangxi Ganyue Expressway Engineering Co., Ltd., Nanchang 330069, China; 2 School of Civil Engineering and Architecture, East China Jiaotong University, Nanchang 330013, China

**Keywords:** hot recycled asphalt mixture, RAP, degree of blending (DOB), low-temperature performance, fatigue lifetime, prediction model

## Abstract

This paper selected three kinds of AC-20 hot-mix recycled asphalt mixtures with high RAP content (30%, 40%, and 50%). It obtains a mixture of different degrees of miscibility by changing RAP preheating temperatures and mixing temperatures. The calculation formula of the degree of blending (DOB) of RAP asphalt interface recycling is proposed. The DSR test quantitatively characterized the DOB mixture’s low temperature, and fatigue properties were tested by beam bending test and four-point bending fatigue test. The prediction models of the recycled mixture’s low temperature and fatigue properties were proposed. The RAP preheating temperature is the most critical factor that dominates both transfers of RAP asphalt to the surface of new aggregate and the effective blending of old and new asphalt. DOB has a significant great influence on low-temperature performance and fatigue performance. The DOB of recycled asphalt can be improved by adjusting and optimizing the process parameters of plant hot recycled mixture to effectively improve the recycled mixture’s low-temperature crack resistance and fatigue lifetime. The optimal RAP dosage and mixing process of required performance can be obtained based on the prediction models to save experimental time and cost.

## 1. Introduction

Hot central plant recycling refers to a maintenance technology, which treats the waste materials of maintenance pavement by milling, recycling, crushing, filing, and so on. It then adds them to the new asphalt mixture in proportion to remix, pave, and compact into the asphalt pavement [1,2]. The hot central plant recycling technology has advantages, such as controllable quality, mature technology, etc., which are widely applied in road engineering [3,4]. It is a maintenance technology with a relatively wide application range and good performance of recycled pavement in all recycling methods [5].

In the hot mix recycled asphalt mixture, the old asphalt wrapped outside the RAP material will be miscible with the added new asphalt in the mixing process, and the degree of miscibility between the two determines the content and properties of effective asphalt in the mixture, thus affecting the pavement performance of the mixture [6]. In the National Cooperative Highway Research Program [7] report, the degree of miscibility between old and new asphalts is defined as completely insoluble (black stone state), partially soluble, and completely soluble. The most acceptable and innovative theory about the miscibility of old and new asphalt is the “partially miscible” theory [8,9]. The core idea of this theory is that in all the old asphalt wrapped on the surface of RAP old aggregate during the process of mixing, transportation, paving, and rolling of recycled asphalt mixture, only the outer part of the old asphalt which is far away from the surface of the old aggregate is blended with the new asphalt. In contrast, the inner layer of the old asphalt which is close to the surface of the old aggregate is still a part of the old aggregate. The inner layer of the old asphalt plays a role, together with the old aggregate, as the skeleton of the recycled mixture, without participating in the mixing of the old and new asphalt, that is, the partial miscible state [10]. Many studies have shown that when RAP content is too high, the two will be in a partially mixed state [7]. Therefore, quantitatively characterizing the mixing state of old and new asphalts in recycled mixture under high RAP blending ratio is the primary key issue to proposing the design method of hot recycled asphalt mixture with high RAP content [11].

Currently, the evaluation indexes of the interfacial blending degree are mostly the conventional performance indexes of the asphalt obtained by extraction test and rotary evaporation test [12,13], such as penetration, softening point, low-temperature flexibility, rheological properties of Brookfield, viscosity, and so on. The test results collected by testing these conventional performance indexes are often relatively discrete, which further indirectly results in the calculated value of the degree of blending (DOB) seriously deviating from the actual value so as not to facilitate quantitative characterization. The creep stiffness modulus S and the curvature of the m value measured by the low-temperature bending beam rheometer (BBR) are not very sensitive under low-temperature test conditions, but the changing range of test results is extremely limited [14], which thereby results in the accuracy of the final test results being difficult to be effectively guaranteed. Due to the small amount of asphalt required for the dynamic shear rheological test (DSR) and the relatively high accuracy of the data collected from the test [9,15], it can be considered for the calculation of the degree of blending (DOB) of interface recycling.

In highway maintenance engineering, the proportion of used materials in the plant-mixed hot recycled mixture is generally not more than 30% [16,17]. The high content of recycled asphalt pavement will cause a serious decline in some road performance, especially the low-temperature crack resistance and durability of the mixture. The low-temperature crack disease is more likely to occur at the early stage of pavement, and then with the repeated fatigue of load, cracks gradually expand to form larger cracks [18], which overweighs the economic benefits obtained from saving materials [19]. Therefore, in order to improve the low-temperature cracking resistance of recycled asphalt mixture, reduce the occurrence of low-temperature cracks in the recycled pavement, improve the using quality of hot recycled asphalt pavement, and prolong the service life of the road, it is necessary to research the low-temperature cracking resistance of hot recycled asphalt pavement [20,21]. In addition, in the preparation of recycled mixture with high RAP content, the change of mixing process will largely affect the mixing state of old and new asphalt in the recycled mixture, thus affecting the road performance of the recycled mixture. At present, there is little research on the influence of interface regeneration and fusion degree of old and new asphalt on road performance of hot recycled asphalt mixture, especially the influence of interface regeneration and fusion degree on low-temperature performance and fatigue life of recycled mixture under high content. Therefore, it is another crucial problem to put forward the design standard of high RAP content recycled asphalt mixture road performance to evaluate the influence of different mixing states of new and old asphalt on road performance.

Given the above two fundamental problems, this paper proposes a mathematical calculation method to characterize the interface degree of blending (DOB) by designing a three-factor and three-level test scheme and calculating the DOB under different RAP content, RAP preheating temperature, and mixing temperatures. The influence of various factors on the interface DOB is analyzed. The low-temperature and fatigue performance of recycled mixture under different mixing processes are studied, and the prediction formulas are proposed. This provides a scientific basis for improving the design method of high RAP content hot recycled asphalt mixture and the promotion and application of plant mixing hot recycling technology on the highway.

## 2. Materials and Methods

### 2.1. Materials

#### 2.1.1. Asphalt

The new asphalt used in this paper during the experimental research is Sinopec Donghai brand SBS-modified asphalt, of which the brand number is I-D. The selected RAP is SBS modified asphalt mixture milled from the municipal roads in Nanchang City. As stipulated in “Technical Specifications for Construction of Highway Asphalt Pavement” (JTG F40-2004), conventional properties of SBS-modified asphalt are tested. Due to the severe aging of old asphalt, the viscosity test at 60 °C cannot be carried out, so the viscosity at 135 °C on it is tested instead. The quality test results and technical indexes of the conventional properties of SBS-modified asphalt are shown in Table 1.

#### 2.1.2. Aggregate and Filler

Limestone is selected as the new aggregate, and the properties of the new and old aggregates are tested according to “Test Methods of Aggregate for Highway Engineering” (JTG E42-2005). The technical indexes are shown in Table 2.

#### 2.1.3. Regenerant

In this paper, combined with practical engineering experience, the RA series thermal regeneration agent is selected, and the dosage of the regeneration agent is 8% of the weight of the total binder required for the mixture. According to the “Technical specification for highway asphalt pavement regeneration” (JTG F41-2008), the performance of the regeneration agent is tested, and the results are shown in Table 3.

#### 2.1.4. Mineral Gradation

**Figure 1:**
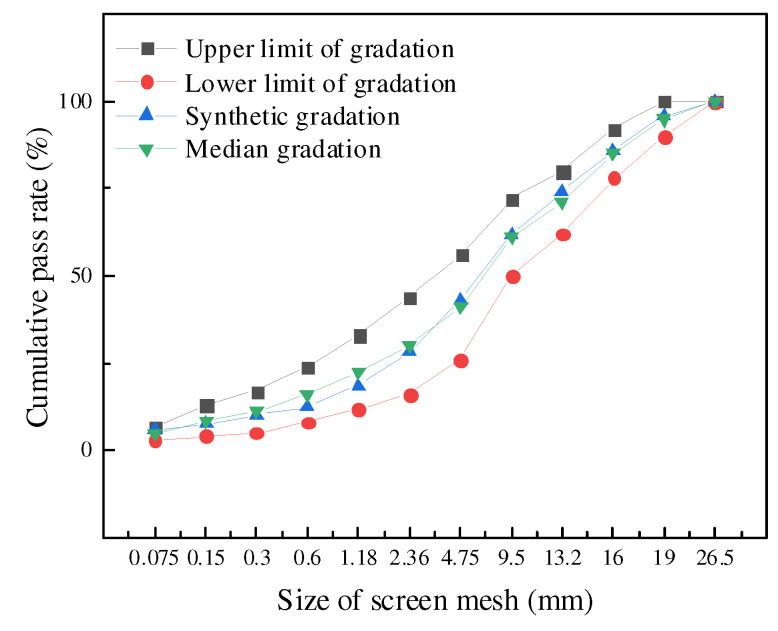
Mineral gradation curves.

In this paper, the recycled mixture with each RAP content (30%, 40%, and 50%) is graded with the same mineral aggregate. On the one hand, the influence caused by different gradations is excluded. In addition, the content of old asphalt in RAP materials is guaranteed to be a single variable, which is convenient for the comparative analysis of various properties of the recycled mixture. Relying on the actual needs of the project, according to the mineral grading range of the targeted AC-20 asphalt mixture, and combined with practical engineering experience, the synthetic gradation of minerals is determined, and gradations are as shown in Figure 1.

### 2.2. Methods

#### 2.2.1. DOB Theory

In this paper, based on the “partial miscible” theory, in consideration of the change of complex shear modulus before and after the blending of old and new asphalts, and aiming at the problem that the degree of interaction and blending on the interface among old RAP asphalt, new asphalt, and regenerant cannot be quantitatively characterized in the recycling process, the evaluation indexes of interfacial recycling and blending of RAP asphalt, i.e., Degree of Blending (DOB), is adopted. The specific formula is shown in Equation (1).

(1) $$\mathrm{DOB}=\frac{G_{new}-G_{old}}{G_{old}-G_{design}}$$

In the Equation, *G_new_* refers to the complex shear modulus of asphalt wrapped on the surface of new aggregate; *G_old_* refers to the complex shear modulus of asphalt wrapped on the surface of old aggregate; *G_design_* refers to the complex shear modulus of asphalt in the recycled mixture.

#### 2.2.2. DOB Test

**Figure 2:**
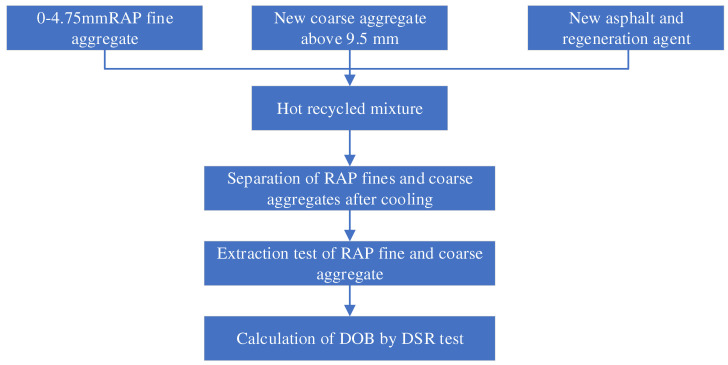
Diagram of DOB test scheme.

To avoid the unfavorable situation that the residual cohesion of RAP fines is too large to form a large-size particle cluster, which cannot effectively distinguish RAP and new aggregates, 4.75 mm is used as the dividing sieve. The RAP fines with particle size below 4.75 mm are selected for the old aggregate, and the new coarse aggregate with a particle size above 9.5 mm is selected for the new aggregate. This prevents the residual cohesive force of RAP fine particles from being too large to form false aggregates with large particle sizes, thus the unfavorable conditions that RAP fine particles and new aggregates cannot be effectively separated are avoided. The DOB test scheme is shown in Figure 2. RAP heating time is 2 h, new asphalt heating temperature is 165 °C, the new aggregate heating temperature is 180 °C, and stirring time is 3 min.

Trichloroethylene solution is used to carry out the extraction and rotary evaporation tests of recycled asphalt wrapped on the surface of new and old aggregates, respectively. Then, the DSR test is carried out on the extracted asphalt and the asphalt in the recycled mixture to obtain the complex shear moduli *G_new_*, *G_old_*, and *G_design_* of three kinds of asphalt.

The RT-DSR 200 series dynamic shear rheometer is used in the test, and the strain control mode is adopted. The test temperature is 64 °C, and the loading frequency is 10 Hz. The asphalt sample is applied by sinusoidal dynamic shear strain with a strain of 12%. The thickness of the asphalt sample is 2 mm, and the main shaft is 8 mm.

The 100% DOB recycled asphalt mixture is prepared to completely extract the old asphalt from the RAP material to be mixed, so that the old asphalt in RAP is wholly separated from the old aggregate, and then the recycled agent, new asphalt, and new aggregate are added to remix. The preheating temperature of RAP is 120 °C, the mixing temperature is 165 °C, and other mixing parameters are unchanged. At this point, the new and old asphalt in the mixture are thoroughly mixed.

#### 2.2.3. Beam Bending Test

According to “Standard Test Methods of Bitumen and Bituminous Mixtures for Highway Engineering” (JTJ052-2000), the rut plate of 300 mm × 300 mm × 50 mm is formed by using the wheel rolling method, and then cut into the prismatic beam specimens of 250 mm × 30 mm × 35 mm. The flexural–tensile failure strain and the beam flexural–tensile strength are tested is tested by using UTM-25 equipment at the test temperature of −10 °C and the loading rate of 50 mm/min.

#### 2.2.4. Four-Point Bending Fatigue Test

According to the “Standard Test Methods of Bitumen and Bituminous Mixtures for Highway Engineering” (JTJ052-2000), the specimens were first formed by a shear compaction instrument, and then cut into 380 mm × 63.5 mm × 50 mm middle beam specimens. Then, three strain levels of 200 με, 400 με, and 600 με were used. The test temperature was 15 °C, the loading frequency was 10 Hz, and the continuous sinusoidal loading mode was applied. Finally, the four-point bending fatigue test was carried out by UTM-25.

## 3. Results and Discussion

### 3.1. DOB

Recycled asphalt mixture contains a certain proportion of RAP, and hot recycling conditions mainly include RAP content, RAP preheating temperature, and RAP mixing temperature. The difference in the performance of recycled mixture under different recycling conditions is mainly caused by the difference in the blending degree of old and new asphalt. The RAP preheating temperature, the mixing temperature, and the content will affect the blending of old and new asphalt in recycled asphalt mixture. Appropriate recycling conditions can improve the blending degree of old and new asphalt to improve the performance of the recycled asphalt mixture.

In order to quickly select the construction parameters with high degree of fusion between new and old asphalt from various horizontal combinations, this paper uses three factors and three levels of orthogonal tests to test RAP asphalt interface recycling mixture. The table of factors and levels is shown in Table 4.

The DOB is calculated and analyzed according to the DSR test results, and the table of DOB test results is then obtained (see Table 5). The DOB results are statistically analyzed, and the average values and the range table of DOB under various hot recycling conditions are then obtained (see Table 6). The trend diagram of DOB results, as shown in Figure 2, is obtained according to factors and levels in Table 6.

From Table 6, it can be determined that among the three factors affecting the degree of blending of RAP asphalt interface recycling, the range value under the factor of the RAP content is 26.8, the range value under the factor of the RAP preheating temperature is 27, and the range value under the factor of the RAP mixing temperature is 5.3, which indicates that the RAP preheating temperature has the most significant influence on the degree of blending of RAP asphalt interface recycling. The influence of various factors on the degree of blending of RAP asphalt interface recycling from the highest to the least is RAP preheating temperature > RAP content > mixing temperature, fully showing that the RAP preheating temperature is the most essential factor leading to the effective blending of old and new asphalt.

**Figure 3:**
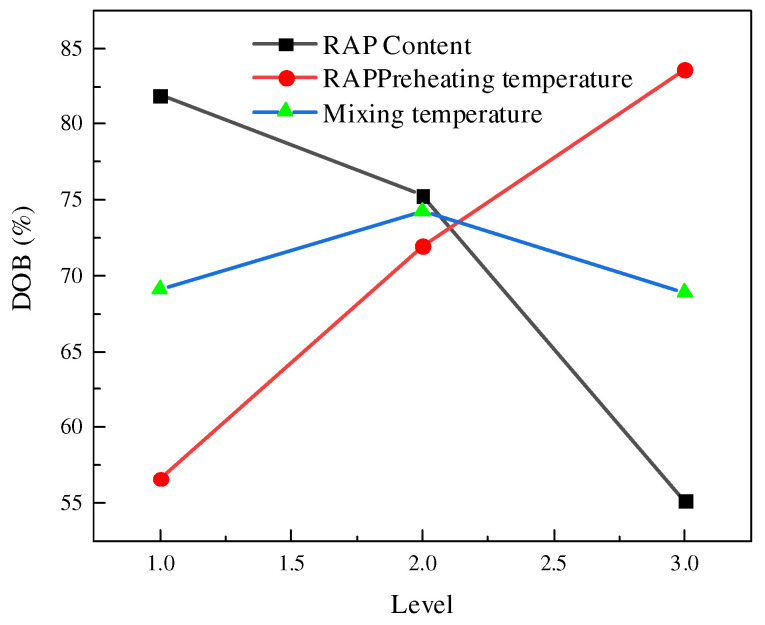
Levels and DOB results changing trend.

It can be seen from Figure 3 that DOB continues to decrease with the increase of RAP content, indicating that if the RAP content is higher, then the older asphalt in the mixture cannot be thoroughly mixed with new asphalt and regenerant, which reduces the cementation between asphalt and aggregate. The reasons lie in the fact that with the increase of RAP content in the recycled mixture, during the whole mixing stage of hot central plant recycling, the old asphalt wrapped on the surface of RAP old mineral material is forced to absorb more material energy required in freeing itself from the bondage of the residual cohesion of the original old mineral material. Therefore, under the same mixing process for the recycled asphalt mixture with different RAP contents, the proportion that effectively activates the old asphalt wrapped on the RAP mineral materials will inevitably vary to varying degrees, further directly leading to the difference in the DOB calculation results. Therefore, the effective recycling rate of the old asphalt decreases instead. An appropriate increase in the mixing temperature can increase the DOB, but if it continues to increase, it will decrease the DOB. The greater the RAP preheating temperature is, the greater the DOB is. However, the excessively high RAP preheating temperature and mixing temperature will aggravate the secondary aging of old asphalt and also lead to the loss of volatilization by heating regenerant. The excessively high heating temperature of new aggregate may also deteriorate the mechanical properties of the new aggregate itself.

In the laboratory, during the mixing and actual production process of the hot recycled mixture, it is necessary to add a certain amount of new asphalt. Then, the old and new asphalt and regenerant (if necessary) infiltrate, diffuse, invade, interact, and blend to form blending recycled asphalt, and finally achieve the purpose of effectively recycling aging asphalt. In order to activate the old asphalt in RAP old material to the maximum extent as much as possible and effectively improve the degree of blending of RAP asphalt interface recycling, the preheating temperature of RAP should be appropriately increased, and the mixing temperature should be reasonably increased under the appropriate RAP content.

### 3.2. Low-Temperature Performance of RAP Mixture

The beam bending test of recycled asphalt mixture produced under preset mixing process parameters is carried out using the universal testing machine. The test results are shown in Table 7.

**Figure 4:**
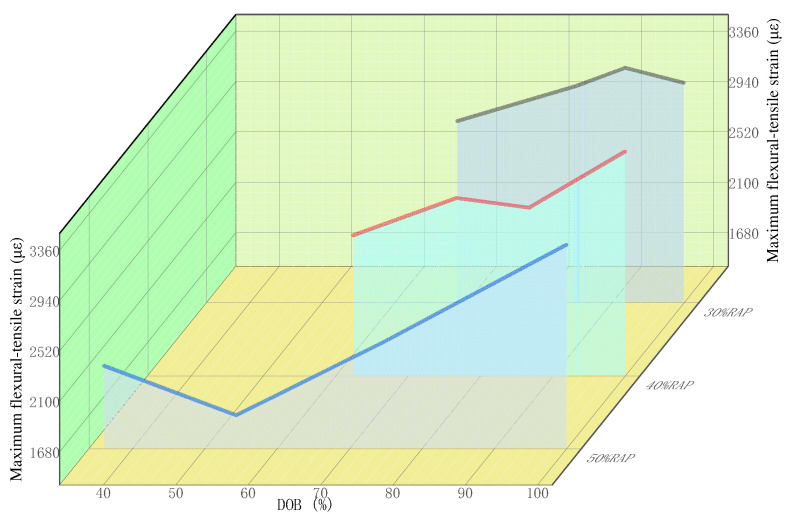
Variation of maximum flexural–tensile strain with the variation of DOB under different RAP contents.

**Figure 5:**
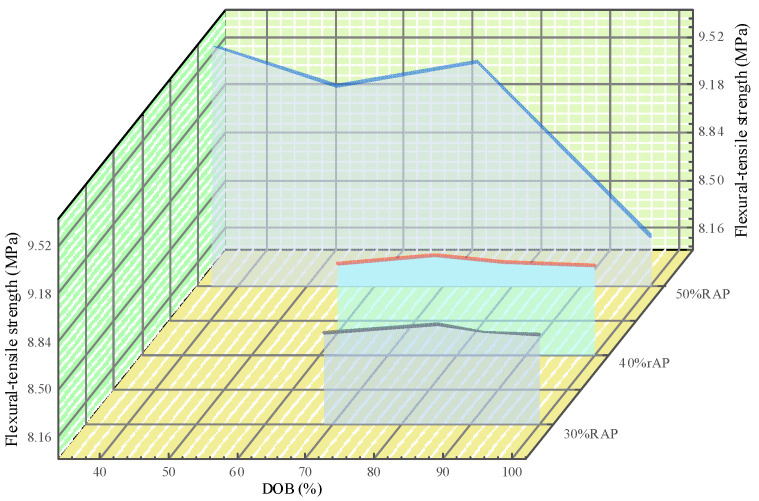
Variation of flexural–tensile strength with the variation of DOB under different RAP contents.

According to the test results in Table 7, the variation of maximum flexural-tensile strain and flexural-tensile strength of recycled mixture with the variation of DOB under different RAP contents are plotted, as shown in Figure 4 and Figure 5.

From Table 7 and Figure 4, it can be determined that in the low-temperature beam test of the recycled mixture under three RAP contents, i.e., 30%, 40%, and 50%, the overall trend of the maximum flexural–tensile strain of the mixture increases by varying degrees with the increase of DOB. The higher the RAP content, the greater the maximum flexural–tensile strain’s growth rate. The reasons lie in the fact that when the miscibility state of old and new asphalt increases, the old asphalt in the recycled mixture is continuously stripped off from the surface of the old aggregate and then transformed into free asphalt. After being miscible with the new asphalt and regenerant, the old asphalt can restore to the new asphalt’s performance standard to enhance the mixture’s overall tenacity, so it shows the phenomenon that the maximum flexural–tensile strain constantly increases. The test results indicate that the maximum flexural–tensile strains of the recycled mixture with 30% and 40% RAP contents are greater than the lower limit of the maximum flexural–tensile strain (2500 με) specified in the current asphalt pavement regeneration technical specification for the winter cool zone, that is, all the low-temperature cracking resistance performance of the recycled mixture is qualified. It indicates that compared with the ordinary hot mix asphalt mixture, the hot central plant recycled mixture with high RAP content (the content of the old material is not more than 40%) can also serve the actual engineering projects. However, the maximum flexural–tensile strains do not meet the specification requirements for three kinds of the recycled mixture under the partial blending states of 50% RAP content. The reasons lie in the fact that under the long-term effect of load and environment, asphalt of RAP material becomes brittle and hard with reduced plasticity and is likely to fracture under low-temperature conditions.

It can be seen from Figure 5 that the flexural–tensile strength of recycled mixture shows a decreasing trend in varying degrees with the increase of the degree of blending (DOB) of RAP asphalt interface recycling. Specifically, with the gradual increase of RAP content, the decreasing amplitude of the flexural–tensile strength is more prominent. The reason is that with the continuous increase of free asphalt, the plasticity of the overall asphalt in the recycled mixture increases, which leads to a decrease in the overall strength of the mixture. Therefore, the flexural–tensile strength thereof decreases with the increase of the miscibility state.

By observing the curve ends in Figure 4 and Figure 5, it is not difficult to find that when the degree of blending (DOB) of RAP asphalt interface recycling reaches the situation of complete blending, the maximum flexural–tensile strain and flexural–tensile strength of recycled mixture under different RAP contents tend to be basically consistent. The reason is that the degree of blending (DOB) of RAP asphalt interface recycling in the recycled mixture gradually increases under the combined effect of more excellent mixing process parameters and other factors, which results in that a larger proportion of aging asphalt in the RAP is heated and softened. Then, it gradually freed itself from the bondage of RAP mineral under the effect of mechanical stirring, transformed into active asphalt, and then further produced a greater degree of blending effect with new asphalt and regenerant (if necessary), which promotes the engineering performance of aging asphalt to be closer to the level of original asphalt and further strengthens the overall tenacity of the recycled mixture. Therefore, the maximum flexural–tensile strain of recycled mixture shows a continuously increasing trend. On the contrary, the proportion of active asphalt in the recycled asphalt mixture gradually increases, resulting in the plastic physical characteristics of its structural asphalt film being further strengthened, so that decay of the strength characteristics of the recycled mixture deteriorated. Finally, the flexural–tensile strain decreases with the increased degree of blending (DOB) of RAP asphalt interface recycling.

In summary, with the increase of DOB, the low-temperature performance of the mixture is improved. According to the requirements of the current specifications, any RAP content below 40% can meet the low-temperature performance requirements of the mixture. Therefore, given the situation that the RAP content is greater than 40%, it can be considered to take specific technical measures to adjust and optimize the mixing process parameters of hot central plant recycling, to promote the degree of blending (DOB) of RAP asphalt interface recycling to a certain extent. At this time, a slight increase in the degree of blending (DOB) of the old and new asphalt interface recycling can also play a significant role in recovering and improving the performance of the low-temperature cracking resistance of the recycled mixture.

### 3.3. Fatigue Performance of RAP Mixture

**Figure 6:**
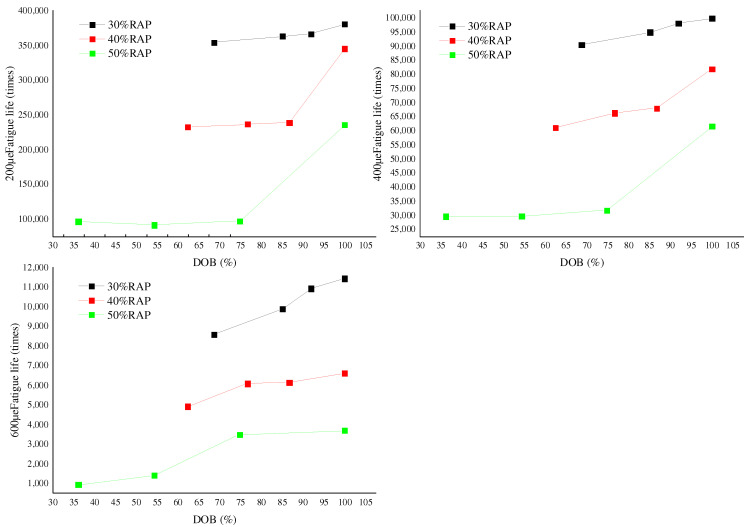
Variation of fatigue lifetimes with the variation of DOB under different RAP contents.

For the recycled asphalt mixture produced under preset mixing process parameters, the four-point bending fatigue test was carried out by using the UTM-25 servo-hydraulic multifunctional material test system. The test results are shown in Table 8. The strain modes are 200, 400, and 600 με, respectively. According to the test results in Table 8, the changes in fatigue life of recycled mixture with DOB under different RAP contents are plotted, as shown in Figure 6.

It can be seen from Table 8 that under the same strain conditions, the fatigue times of each recycled mixture gradually increase with the increase of the mixing state of old and new asphalts. With the increase of RAP preheating temperature and mixing temperature, the fatigue times of the mixture also increase. Especially when the RAP content reaches 50%, the fatigue times of the mixture increase obviously, indicating that increasing the RAP preheating temperature and mixing temperature can improve the fatigue lifetimes of the recycled mixture. This is because the increase in the mixing state increases the content of free asphalt in the mixture and improves the adhesion of asphalt to aggregate, indicating that the increase in the mixing state of old and new asphalt has improved the fatigue lifetimes of the mixture.

It can also be seen from Figure 6 that when the RAP content is higher, the fatigue number of the mixture decreases more obviously, indicating that the effect of RAP content on the fatigue lifetimes of the recycled mixture is more obvious. The fatigue number of the 50% RAP content mixture at 200 με is significantly lower than that of 30% RAP content, and the greater the strain, the more obvious the decrease. This is because the increase in RAP content leads to an increase in the content of old asphalt mixed with new asphalt, which reduces the adhesion of asphalt mortar and reduces the toughness of the recycled mixture, thus affecting the fatigue performance of the mixture significantly.

In summary, for the recycled mixture with high RAP content, the mixing state of old and new asphalts in the mixture can be improved as much as possible by changing the mixing process to improve the fatigue performance of the recycled mixture.

### 3.4. Prediction of Low-Temperature and Fatigue Performance of RAP Mixture

#### 3.4.1. Flexural–Tensile Strength

It can be determined from Table 7 that RAP preheating temperature, mixing temperature, and content influence the low-temperature performance of recycled mixture (excluding three groups of data with 100% DOB). This section takes RAP content, the RAP preheating temperature, and mixing temperature as independent variables to analyze the change of flexural–tensile strength of recycled mixture under three factors. From the experimental data, RAP preheating temperature, mixing temperature, and RAP content have no clear linear law on flexural strength, but the interaction of three factors has an impact on flexural strength. Therefore, nonlinear fitting is considered to predict the bending strength. After many attempts, the bending strength is fitted by the Levenberg Marquardt method through Matlab software. The model after multiple fittings is shown in Formula (2).

(2) $$Y_{1}=19.707-71.273x_{1}{}^{-1.02}-\frac{10.007x_{2}{}^{0.061}}{x_{3}{}^{0.082}}$$

In the formula, *Y*_1_ refers to flexural–tensile strength, MPa; *x*_1_ refers to RAP content, %; *x*_2_ refers to RAP preheating temperature, °C; *x*_3_ refers to mixing temperature, °C.

The model correlation coefficient R^2^ = 0.951, indicating that the prediction model can well reflect the changing trend of the flexural–tensile strength of recycled mixture under different RAP contents and different construction technologies. It can be determined from Formula (2) that the higher the RAP content and the mixing temperature are, the greater the flexural–tensile strength of the recycled mixture is. The higher the RAP preheating temperature, the smaller the compressive strength, but the overall effect is relatively weak.

#### 3.4.2. Maximum Flexural–Tensile Strain

This section takes RAP content, RAP preheating temperature, and mixing temperature as independent variables to analyze the change of maximum flexural–tensile strain of the recycled mixture under the influence of three factors. Since the three factors have no clear linear influence rules on the maximum flexural–tensile strain, the nonlinear model is also used for fitting. The model after multiple fittings is shown in Formula (3).

(3) $$Y_{2}=1831.5-0.0034x_{1}{}^{3.306}+3.2833\times 10^{-9}x_{2}{}^{5.144}+2.52x_{3}{}^{1.236}$$

In the formula, *Y*_2_ refers to flexural-tensile strength, με; *x*_1_ refers to RAP content, %; *x*_2_ refers to RAP preheating temperature, °C; *x*_3_ refers to mixing temperature, °C.

The correlation coefficient of the model R^2^ = 0.973 indicates that the prediction model can well reflect the changing trend of the maximum flexural–tensile strain of recycled mixture under different RAP contents and different construction technologies. It can be determined from Formula (3) that the higher the RAP preheating temperature and the mixing temperature are, the larger the maximum flexural–tensile strain of the recycled mixture is.

#### 3.4.3. Fatigue Lifetime

In this section, the change of fatigue life of the recycled mixture under 200 με, 400 με, and 600 με strains was analyzed under the influence of three factors, including RAP content, RAP preheating temperature, and mixing temperature. The nonlinear model is used for fitting, and the model after multiple fitting is shown in Formulas (4)–(6). Formulas (4)–(6) are fatigue life prediction models under the strain of 200 με, 400 με, and 600 με, respectively.

(4) $$L_{200}=52490-1558.4x_{1}{}^{1.477}+3087.605x_{2}{}^{0.259}x_{3}{}^{0.376}$$

(5) $$L_{400}=119540-153.46x_{1}{}^{1.683}+161.97x_{2}{}^{0.974}+0.033x_{3}{}^{2.32}$$

(6) $$L_{600}=15220-835.53x_{1}{}^{0.841}+7.179x_{2}{}^{1.348}+115.96x_{3}{}^{0.721}$$

In these formulas, *L* refers to fatigue lifetime, times; *x*_1_ refers to RAP content, %; *x*_2_ refers to RAP preheating temperature, °C; *x*_3_ refers to mixing temperature, °C.

The correlation coefficients R^2^ of models (4)–(6) are 0.999, 0.999, and 0.994, respectively, indicating that these three fatigue prediction models can well reflect the variation trend of fatigue life of the recycled mixture under different RAP contents and different construction technologies. These prediction models will be different due to different materials such as asphalt, RAP, and regenerant. However, the test and prediction methods are similar, and factors such as mixing time and mixing temperature can also be included in the model in the follow-up study.

According to these prediction models, in practical engineering applications, if the RAP content is determined, the bending tensile strength, bending tensile strain, and fatigue lifetime change diagrams can be plotted at the RAP preheating temperature and mixing temperature. Based on this diagram, the most suitable RAP preheating and mixing temperature can be obtained by comprehensively considering the economic cost and actual performance, thus saving much test time and cost.

## 4. Conclusions

In this paper, an experimental scheme of three factors and three levels is designed, and a mathematical calculation method to characterize the interface regeneration and fusion degree is proposed. The interface regeneration and fusion degree were analyzed under different RAP content, preheating, and mixing temperatures. Through the beam bending and four-point bending tests, the low-temperature and fatigue performance of recycled asphalt mixture under different conditions is evaluated, and its low-temperature and fatigue performance are predicted. The main conclusions are as follows.

(1) The influence on the DOB of RAP asphalt interface recycling from the highest to the least is in the order of RAP preheating temperature > RAP content > mixing temperature. The RAP preheating temperature is the most critical factor that dominates the effective blending of old and new asphalt. In order to activate the old asphalt in RAP old materials to the maximum extent possible and effectively improve the degree of blending of RAP asphalt interface recycling, the RAP preheating temperature should be appropriately increased, and the mixing temperature should be reasonably increased under the appropriate RAP content.

(2) The overall trend of the maximum flexural–tensile strain of recycled mixture is that it increases to varying degrees with the increase of DOB. In contrast, the flexural-tensile strength decreases with increased RAP asphalt interface DOB. When the RAP asphalt interface DOB reaches the complete fusion situation, the recycled mixture’s maximum flexural strain and flexural strength under different RAP content are basically consistent. Under the same strain condition, the fatigue lifetime of each recycled mixture increases gradually with the increase of the mixing state of old and new asphalt. The influence of RAP content on the fatigue lifetime of the recycled mixture is relatively apparent, and the greater the strain is, the more pronounced the reduction is. With the increase of RAP preheating temperature and mixing temperature, the fatigue lifetime of the mixture increases.

(3) For the recycled mixture with high RAP content, it can be considered to take specific technical measures to adjust and optimize the process parameters of plant mixing the hot recycled mixture, and improve the DOB of RAP asphalt, to improve the low-temperature performance and fatigue performance of recycled mixture. According to the low-temperature performance and fatigue performance prediction model, in practical engineering applications, if the RAP content is determined, the bending tensile strength, bending tensile strain, and fatigue life change diagram can be drawn by RAP preheating temperature and mixing temperature. According to the diagram, the economic cost and actual performance can be comprehensively considered to obtain the most suitable RAP preheating temperature and mixing temperature to save much test time and cost.

## Figures and Tables

**Table 1:** Test results of conventional indexes of new and old asphalt.

Categories of Materials	25 °C Penetration (0.1 mm)	5 °C Ductility (cm)	Softening Point (°C)	135 °C Viscosity (Pa·s)
Old asphalt	26.9	brittleness	87.2	16.8
New asphalt	58.6	27	73.5	2.9
SBS modified asphalt (I-D) Standard requirements	40~60	≥20	≥60	<3

**Table 2:** Conventional technical indexes of new and old aggregates.

Categories of Materials	Crushed Value (%)	Apparent Density (g·cm^−3^)	Water Absorption (%)	Water Washing Treatment <0.075 mm (%)	Flat and Elongated Particle Content (%)
Old aggregate	16.7	2.715	1.2	0.8	5.5
New aggregate	16	2.713	0.474	0.71	9.7
Standard requirements	≤26	≥2.6	≤2	<5	≤15

**Table 3:** Property indexes of regenerant.

Categories of Materials	60 °C Viscosity (mPa·s)	Flashpoint (°C)	Saturates Content (%)	Aromatics Content (%)	Viscosity Contrast before and after Thin Film Oven Test (%)	Quality Changes before and after Thin Film Oven Test (%)	Density (g·cm^−3^)
Regenerant	372.4	263	22.8	42.98	2.48	−3.17	1.013
Standard requirements	176~900	≥220	≤30	measured	≤3	≤4, ≥−4	measured

**Table 4:** Factors and levels of orthogonal test.

	Factors	RAP Content (%)	RAP Preheating Temperature (°C)	Mixing Temperature (°C)
Levels				
Level 1		30	100	150
Level 2		40	120	165
Level 3		50	140	180

**Table 5:** DOB test results.

RAP Content (%)	RAP Preheating Temperature (°C)	Mixing Temperature (°C)	DOB (%)
30	100	150	68.7
30	120	165	85.1
30	140	180	91.9
40	100	165	62.4
40	120	180	76.7
40	140	150	86.7
50	100	180	36.1
50	120	150	54.3
50	140	165	74.8

**Table 6:** Range analysis results.

Analysis Value	Factors		
	RAP Content (%)	RAP Preheating Temperature (°C)	Mixing Temperature (°C)
$\bar{K}1$	81.9	56.6	69.1
$\bar{K}2$	75.3	72.0	74.2
$\bar{K}3$	55.1	83.6	68.9
R	26.8	27	5.3

**Table 7:** Test results of the low-temperature beam bending test.

RAP Content (%)	RAP Preheating Temperature (°C)	Mixing Temperature (°C)	DOB (%)	Maximum Flexural–Tensile Strain (με)	Flexural–Tensile Strength (MPa)
30	100	150	68.7	2915	8.65
30	120	165	85.1	3207	8.71
30	140	180	91.9	3359	8.66
30	120	165	100	3235	8.64
40	100	165	62.4	2568	9.49
40	120	180	76.7	2880	9.15
40	140	150	86.7	2798	9.07
40	120	165	100	3269	8.75
50	100	180	36.1	2086	9.71
50	120	150	54.3	1673	9.42
50	140	165	74.8	2288	9.59
50	120	165	100	3098	8.35

**Table 8:** Test results of the four-point bending fatigue test.

RAP Content (%)	RAP Preheating Temperature (°C)	Mixing Temperature (°C)	DOB (%)	200 με Fatigue Lifetime (Times)	400 με Fatigue Lifetime (Times)	600 με Fatigue Lifetime (Times)
30	100	150	68.7	354,216	90,349	8573
30	120	165	85.1	363,048	94,817	9894
30	140	180	91.9	366,499	98,006	10,917
30	120	165	100	380,437	99,854	11,425
40	100	165	62.4	232,458	61,063	4919
40	120	180	76.7	236,661	66,205	6094
40	140	150	86.7	238,978	67,946	6144
40	120	165	100	345,267	81,766	6627
50	100	180	36.1	96,527	29,559	926
50	120	150	54.3	91,389	29,737	1414
50	140	165	74.8	96,944	31,749	3482
50	120	165	100	235,818	57,510	3695

## Data Availability

Data is contained within this article.
